# Account of Patients Admitted from July 1, to Aug. 1, 1801

**Published:** 1801-09

**Authors:** W. White

**Affiliations:** Bath


### *To the Editors of the Medical and Phyſical Journal.*

GENTLEMEN,

I Have taken the liberty of ſending you an account of the caſes admitted at the Bath City Diſpenſary, in the courſe of the laſt month, if you think it worth a place in your valuable Publication. I am, &c.

*Bath, Aug.* 8, 1801. W. WHITE.

---

From the following ſtatement, it will be found that the Synochus Bilioſa is the moſt prevalent diſeaſe we have at preſent in this city; and as it has been attended with ſome peculiar circumſtances hitherto unnoticed, I hope to have in my power ſoon to communicate to the public a ſhort hiſtory of it.

*Account of Patients admitted from July* 1, *to Aug.* 1, 1801.

| | | | |
|---|---|---|---|
| Angina | 2 | Pulmonary Compl. | 1 |
| Aſthma | 1 | Prolapſus Ani | 1 |
| Anaſarca | 1 | ——— Uteri | 1 |
| Catarrhus Febrilis | 1 | Pſora | 7 |
| Chloroſis | 2 | Rheumatiſm Acute | 10 |
| Contuſion | 1 | Rachitis | 1 |
| Eruptiones | 3 | Synochus Bilioſa | 38 |
| Febris Simplex | 4 | ——— Quotidiana | 1 |
| ——— Quotidiana | 1 | ——— Pleuritica | 5 |
| ——— Puerperalis | 1 | ——— Enteritica | 3 |
| Gaſtrodynia | 1 | ——— Gaſtritica | 4 |
| Hydrocephalus | 1 | ——— Rheumatica | 4 |
| Hernia | 2 | ——— Cholerica | 2 |
| Hæmoptyſis | 1 | ——— C. Hemiplegia | 1 |
| Hæmatemeſis | 1 | ——— Nephritica | 1 |
| Herpes | 2 | Syphilis | 4 |
| Icterus | 1 | Sprain | 1 |
| Lepra | 3 | Suppuratio | 1 |
| Menorrhagia | 2 | Strictures in Urethra | 1 |
| Nephritic Compl. | 1 | Tumor in the Groin | 1 |
| Ophthalmia | 1 | Tuſſis | 2 |
| Pleuritis | 1 | Tinea Capitis | 1 |
| Pertuſſis | 2 | Varicoſe Ulcers | 10 |
| Phthiſis | 1 | Variola Diſcreta | 1 |

On my firſt viſiting the perſon attacked with Hemiplegia, I was not clear whether it was an original or ſymptomatic affection, but was afterwards convinced it was the latter, and the complaint a variety of the Synochus Bilioſa. The ſubject of it, it, (a man aged about 40) was ſeized in the morning with giddineſs, dimneſs of ſight, and head-ach; ſucceeded by the loſs of ſpeech, and of the uſe of his right ſide. I did not ſee the patient till very late in the evening; his pulſe was full, ſtrong, and frequent; he could not ſpeak, but appeared under great anxiety. He had vomited ſome green bile. Before I ſaw him, twelve ounces of blood had been taken from his arm, which was highly inflamed; and as his pulſe indicated the neceſſity of repeating the operation, I took away eight ounces more; after which ten grains of calomel were preſcribed, and a ſolution of neutral ſalts to be taken every four hours. Next morning his bowels were moved, and a great quantity of dark bile was evacuated; after which he could move his fingers a little, and the day following perfectly recovered his ſpeech, and the uſe of his arm and leg.

